# Comprehensive Analysis of the Impact of Climate Change and Human Activities on the Distribution of Five *Fritillaria* Species Using the Optimized Maxent Model

**DOI:** 10.1002/ece3.72305

**Published:** 2025-10-13

**Authors:** Yuanyuan Li, Qinghe Wang, Rong Ding, Xiaofen Liu, Sijing Liu, Jing Bai, Shuqi Niu, Jinlin Guo

**Affiliations:** ^1^ State Key Laboratory of Southwestern Chinese Medicine Resources, College of Pharmacy Chengdu University of Traditional Chinese Medicine Chengdu China; ^2^ School of Ethnic Medicine Chengdu University of Traditional Chinese Medicine Chengdu China; ^3^ College of Medical Technology Chengdu University of Traditional Chinese Medicine Chengdu China

**Keywords:** climate change, five *Fritillaria* species, human footprint, Maxent model, suitable area

## Abstract

With climate change and the influence of human activities, species are likely to migrate or even go extinct. Five *Fritillaria* species, a well‐known traditional Chinese medicinal plant, are rarer due to overharvesting. This study employed the Maxent model to identify suitable areas for the plant, determine key environmental factors, and project future shifts under three climate change scenarios. The analysis showed *F. przewalskii* and *F. delavayi* might migrate to higher elevations, while *F. taipaiensis* was expected to move to lower elevations. There were differences in the dominant environmental factors among different origins: *F. cirrhosa* (elevation, bio7, bio9, bio12, hfp); *F. unibracteata* (elevation, bio4, bio15, bio19, hfp); *F. przewalskii* (elevation, bio4, bio11, bio15, hfp); *F. delavayi* (elevation, bio3, bio18, hfp); *F. taipaiensis* (bio2, bio3, bio4, bio11, hfp). Under the SSP585 scenario, the suitable areas of *F. cirrhosa*, *F. przewalskii,* and *F. taipaiensis* were contracting, while those of *F. unibracteata* and *F. delavayi* were rising. Also, the centroids of *F. cirrhosa* and *F. przewalskii* shifted slightly northeastward, *F. unibracteata*'s shifted southward, and *F. delavayi* and *F. taipaiensis*'s shifted northwestward. These findings provide a foundation for the conservation, sustainable management, and cultivation of five *Fritillaria* species.

## Graphical abstract

This study utilized extensive specimen data and detailed geographic and environmental information, applying the Maxent model to predict the distribution of *F. cirrhosae* bulbus. The key findings are as follows: (1) *F. przewalskii* and *F. delavayi* tend to migrate to higher altitudes, while *F. taipaiensis* is shifting to lower altitudes. (2) Among different species of *F. cirrhosae bulbus*, altitude, temperature, and precipitation significantly influence distribution, with altitude being the most critical factor. (3) Under the SSP585 scenario, the suitable areas of *F. cirrhosa*, *F. przewalskii*, and *F. taipaiensis* were contracting, while those of *F. unibracteata* and *F. delavayi* were rising. Also, the centroids of *F. cirrhosa* and *F. przewalskii* shifted slightly northeastward, *F. unibracteata* shifted southward, and *F. delavayi* and *F. taipaiensis* shifted northwestward.

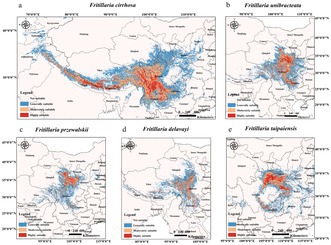

## 1 Introduction

Due to the impact of changes in natural conditions and human activities, global temperatures are trending upward (Nogué et al. 2009). The new report of the Intergovernmental Panel on Climate Change (IPCC) shows that greenhouse gases can cause global warming (Sills 2024). From 2011 to 2020, the global surface temperature was 1.2°C ± 0.1°C higher than that from 1850 to 1900 (Voosen 2021). Climatic conditions are critical determinants of species' natural geographic distribution (Veloz et al. 2011). Climate change has reshaped species distribution and affected biodiversity (Wang et al. 2020), while human activities have further degraded plant diversity through habitat fragmentation and resource exploitation. Recently, many studies use species distribution models to predict climate change's and human activities' impact on species and habitats (Zhang et al. 2024).

Species distribution models (SDMs), also known as niche models, are empirical methods for quantifying the species' occupied environment (Zhang et al. 2018; Li et al. 2020; Wen et al. 2022). These models integrate species distribution data with a set of environmental variables and then project them into geographical space to further predict the potential distribution of species (Elith and Leathwick 2009). SDMs are essential tools in ecology, conservation, evolutionary biology, biogeography, and climate change research (Guisan 2017). They are widely applied to examine the impact of climate change on species distribution (Guo et al. 2019), the conservation of rare and endangered species (Escalera‐Vázquez et al. 2018), species invasion (Bowen and Stevens 2020), and wildlife management (Zhang et al. 2019). At present, commonly used SDMs include the bioclimate analysis and prediction system (BIOCLIM) (Serrano‐Notivoli et al. 2022), ecological niche factor analysis (ENFA) (Hengl et al. 2009), genetic algorithm for rule‐set production (GARP) (Stockwell 1999), and maximum entropy modeling (Maxent) (Huang et al. 2024), etc. Maxent is favored for its stability, ease, small sample need, and accurate predictions (Li et al. 2017). Furthermore, studies show that optimizing parameters can remarkably boost the predictive precision and dependability of the model (Vignali et al. 2020). For example, Zhao et al. (2024) developed an optimized Maxent model to predict the current and future suitable areas for the invasive pest *Lycorma delicatula* worldwide, which provided valuable insights into the risk of introducing *L. delicatula* under both currents. Additionally, Wang et al. (2024) used the optimized Maxent model to analyze the suitable planting areas and climate preference of *Reaumuria songarica*, which provided a reliable basis for targeted desertification control using such desert‐adapted species. Zhu et al. (2024) and Fang et al. (2024) further demonstrated the model's utility in predicting habitat suitability dynamics for the medicinal plants *Pyrethrum tatsienense* and *Cirsium japonicum* Fisch. ex DC., respectively. These collective findings provide references for rational cultivation planning, agricultural system optimization, and climate‐adaptive conservation strategies. Understanding the ecological requirements of five *Fritillaria* species is crucial for its sustainable cultivation and conservation, given its significance in traditional medicine and vulnerability to environmental changes.

*F. cirrhosae* bulbus is a precious Chinese medicinal herb which is the dried bulb of several plants in the genus *F*. of the Liliaceae family (Ma et al. 2021). It has been listed as a national second‐grade key protected wild plant (https://www.iplant.cn/bhzw/), and its legally defined recognized species include *F. cirrhosa*, *F. unibracteata*, *F. przewalskii*, *F. delavayi*, *F. taipaiensis* (National Pharmacopoeia Commission 2020). According to the 2020 edition of Chinese Pharmacopeia, *F. cirrhosae* bulbus is used for clearing heat and moistening the lungs, resolving phlegm and relieving cough, dissipating nodules and treating carbuncles (Liu 2023). As a traditional medicinal plant, the five *Fritillaria* species face severe overexploitation due to their extensive clinical applications. This not only directly diminishes wild populations but also disrupts natural distribution patterns through habitat destruction (Li et al. 2012). Critically, while wild *Fritillaria* specimens exhibit superior bioactive compound concentrations and enhanced expectorant efficacy, cultivated varieties remain indispensable for clinical supply. To reconcile conservation‐clinical demand tensions, standardized cultivation protocols have emerged as a core strategy for sustainable utilization. Identifying the precise environmental requirements of wild five *Fritillaria* species populations provides essential scientific foundations for site selection of cultivation zones, habitat simulation protocols, and quality enhancement initiatives within the cultivation system (Tao et al. 2024). Although previous studies have examined the habitat suitability and future distribution of species such as *F. cirrhosa*, *F. unibracteata*, *F. przewalskii*, and *F. delavayi* (Huang 2023), these studies have been limited by small sample sizes and incomplete species coverage potentially failing to comprehensively address changes in habitat suitability under varying environmental conditions and the broader trends in future distribution.

This study aims to investigate the impact of climate change on the distribution of five *Fritillaria* species. Based on extensive sample data, we simulated future climate change scenarios for five *Fritillaria* species under varying emission concentration scenarios. The specific objectives of this research are as follows: (i) To collect comprehensive specimen data on five *Fritillaria* species and assess the correlation between the elevation of each original source and temporal variation. (ii) To apply the Maxent model to determine the key environmental factors affecting the distribution range of five *Fritillaria* species. (iii) To utilize the ArcGIS system for a detailed analysis of the spatial changes of suitable area, both in the present and under different future development scenarios. The findings of the study will assist in developing strategies for the wild tending and cultivation of five *Fritillaria* species, offer insights for conservation and sustainable use, and lay a foundation for the protection of its wild resources.

## 2 Materials and Methods

### 2.1 Collection and Processing of Specimen Data

**FIGURE 1:**
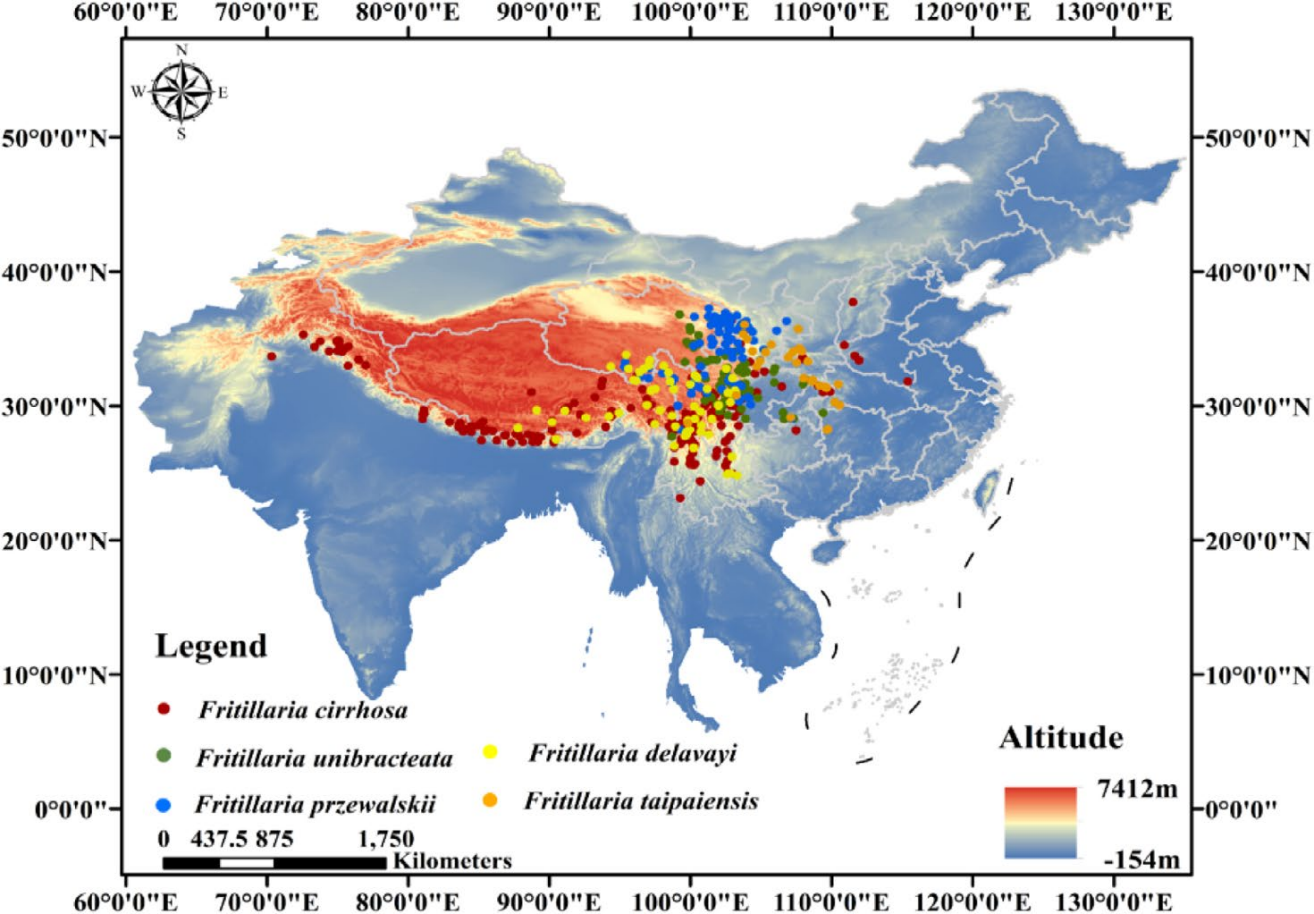
The distribution points of five *Fritillaria* species.

Specimen data for five *Fritillaria* species were primarily sourced from several reputable databases, including the Chinese National Plant Specimen Resource Center (https://www.cvh.ac.cn/), National Specimen Information Infrastructure (NSII) (https://www.nsii.org.cn/), Global Biodiversity Information Facility (GBIF) (https://www.gbif.org/zh/), Integrated Digitized Biocollections (iDigbio) (https://www.idigbio.org/), the Standardized Organization and Resource Sharing Platform for Teaching Specimens (http://mnh.scu.edu.cn/list/sample), and the National Germplasm Resource Bank of Traditional Chinese Medicine (The Fourth National Survey of Traditional Chinese Medicine Resources). The data, including collection time, location, elevation, latitude, and longitude, were organized, with ambiguous, duplicate, or erroneous entries excluded. ENMTools (Environmental Niche Model Tools), a software package designed for ecological niche modeling and species distribution modeling (Warren et al. 2010), was used for data screening to reduce sample bias and overfitting, ensuring one occurrence point per 2.5 km grid (5 km × 5 km). After processing, a total of 547 records remained, mainly distributed across the Qinghai‐Tibet Plateau and adjacent high‐elevation areas, with additional coverage in Southwest China, Northwest China, and parts of the central and eastern regions. Among them, 227 points for *F. cirrhosa*, 115 for *F. unibracteata*, 107 for *F. przewalskii*, 66 for *F. delavayi*, and 32 for *F. taipaiensis* were obtained (Figure 1) and saved in “.csv” format for future use.

### 2.2 Acquisition and Screening of Geographic and Environmental Data

Geographic data were downloaded from the Resource and Environmental Science Data Center (www.resdc.cn), with 19 bioclimatic variables sourced from Worldclim (https://worldclim.org). Three topographic variables (slope, elevation, and aspect) were extracted from elevation data using ArcMap 10.8. Soil data were downloaded from the Harmonized World Soil Database. Human footprint data were obtained from The Earth Science Data Systems (www.earthdata.nasa.gov), which covered eight dimensions: built environment, population density, nighttime lights, cropland, pasture, roads, railroads, and navigable waterways (Mu et al. 2022), totaling 27 environmental factors at a 2.5‐min resolution (Table S1; Dorji 2016; Chen et al. 2022; Rajlaxmi et al. 2024).

Environmental data for 2041–2060 and 2061–2080 were derived from SSP126, SSP245, and SSP585 scenarios based on the BCC‐CSM2‐MR (Beijing Climate Center Climate System Model, version 2, Medium Resolution) model in IPCC6. These represent low, medium, and high greenhouse gas emissions pathways, respectively (Shi et al. 2020; Su et al. 2021). The BCC‐CSM2‐MR, refined for reliability, performs best in East Asia (Guo et al. 2022). To prevent overfitting and reduce accuracy loss from spatial collinearity, pre‐modeling was conducted. A random 25% of the data was used as the test set, while 75% served as the training set. Iterative calculations were performed 1000 times and repeated 10 times. The output was set to logistic, and other parameters were set to default. Jackknife analysis determined the contribution of each environmental variable, and Spearman correlation analysis excluded variables with low contribution and high correlation (|*r*| ≥ 0.8, Figure S1). Finally, 9, 10, 9, 9, and 10 variables were selected for modeling for *F. cirrhosa*, *F. unibracteata*, *F. przewalskii*, *F. delavayi*, and *F. taipaiensis*, respectively (Table 1; Li et al. 2024).

**TABLE 1:** Environmental variables related to the distributions.

Abbreviation	Climate variables	Unit
Bio2	Mean diurnal range	°C
Bio3	Isothermality (bio2/bio7) (×100)	—
Bio4	Temperature Seasonalit (standard deviation × 100)	—
Bio7	Temperature annual range (bio5–bio6)	°C
Bio9	Mean temperature of driest quarter	°C
Bio11	Mean temperature of coldest quarter	°C
Bio12	Annual precipitation	mm
Bio15	Precipitation seasonality (coefficient of variation)	—
Bio18	Precipitation of warmest quarter	mm
Bio19	Precipitation of coldest quarter	mm
Elev	Altitude	m
Slope	Slope	°
Aspect	Aspect	rad
Awc_class	Awc range	code
T_sand	Sand soil content	%
T_oc	Topsoil organic carbon	% weight
T_ph_h2o	Topsoil pH (H_2_O)	−log (H^+^)
T_clay	Topsoil clay fraction	% wt.
Hfp	Human foot print	—

### 2.3 The Optimization and Evaluation of the Maxent Model

Previous research indicates that the Maxent model using default parameters often yields suboptimal predictive performance. Meanwhile, the model performance is substantially influenced by the feature combination (FC) and regularization multiplier (RM). To address this, we employed the ENMeval package for parameter optimization. Six FCs and eight RM values were systematically evaluated through cross‐combination. Parameters settings were subsequently calibrated to maximize model precision based on delta AICc minimization, ensuring optimal predictive performance (Wang et al. 2024; Zhao et al. 2024). The six FCs were L, LQ, H, LQH, LQHP, and LQHPT, which represent linear (L), quadratic (Q), fragmented (H), hinge (P), and threshold (T) features, respectively. The eight RM values ranged from 0.5 to 4, with intervals of 0.5 (Zhao et al. 2024; Zhu et al. 2024). The 48 combinations were tested in ENMeval, and the best one, based on delta.AICc = 0, was selected for Maxent modeling (Muscarella et al. 2014).

The spatial point data for the five selected species, along with the environmental variables, were imported into the Maxent model. For each species, the optimal combinations of FC and RM were chosen, while other variables remained fixed from the pre‐modeling stage. The model's performance was assessed by evaluating its fit to the data using the Receiver Operating Characteristic (ROC) curve. Model accuracy was assessed using the AUC of the ROC curve: 0.9 ≤ AUC < 1.0 indicates excellent accuracy, 0.8 ≤ AUC < 0.9 indicates good accuracy, 0.7 ≤ AUC < 0.8 indicates average accuracy, and 0.5 ≤ AUC < 0.7 indicates poor accuracy (Wang et al. 2024).

### 2.4 Division of Potentially Suitable Areas

The average ASCII output from 10 Maxent runs was visualized and reclassified in ArcGIS 10.8. Prediction values ranged from 0 to 1, with higher values indicating greater species presence likelihood. Suitability was classified as high (0.6, 1), moderate (0.4, 0.6), general (0.2, 0.4), and unsuitable (≤ 0.2) (Chen et al. 2023; Wang et al. 2023; Luo et al. 2024). Subsequently, changes in suitable habitat for five *Fritillaria* species were analyzed across climate models and time periods.

## 3 Results

### 3.1 Correlation Between the Elevation Distribution of Five *Fritillaria* Species and Time

**FIGURE 2:**
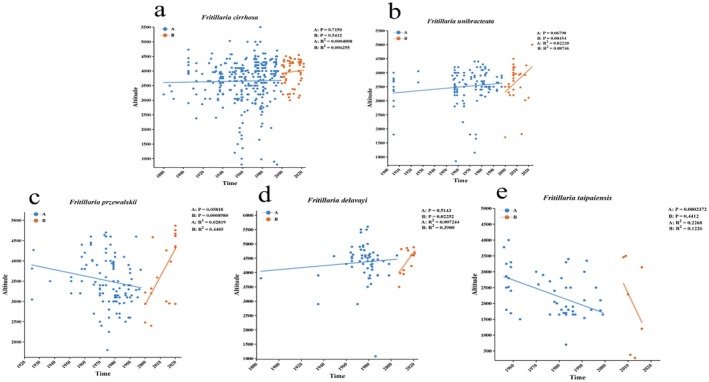
The relationship between elevation and time of five *Fritillaria* species: *F. cirrhosa* (a), *F. unibracteata* (b), *F. przewalskii* (c), *F. delavayi* (d), and *F. taipaiensis* (e).

This study analyzed the elevation‐time relationship for five species using elevation and time data, with 2000 as the demarcation point (Zhao, Chen, et al. 2018). Figure 2 shows that, prior to 2000, only *F. taipaiensis* exhibited a weak elevation‐time correlation (*p* < 0.001, *R* ^2^ = 0.23), while no correlation was observed for other species. After 2000, *F. przewalskii* (*p* < 0.001, *R* ^2^ = 0.45) and *F. delavay*i (*p* < 0.05, *R* ^2^ = 0.39) exhibited weak upward trends in elevation, whereas *F. taipaiensis* showed a downward trend. No correlation was observed for other species post‐2000.

### 3.2 Model Optimization and Accuracy Evaluation

**FIGURE 3:**
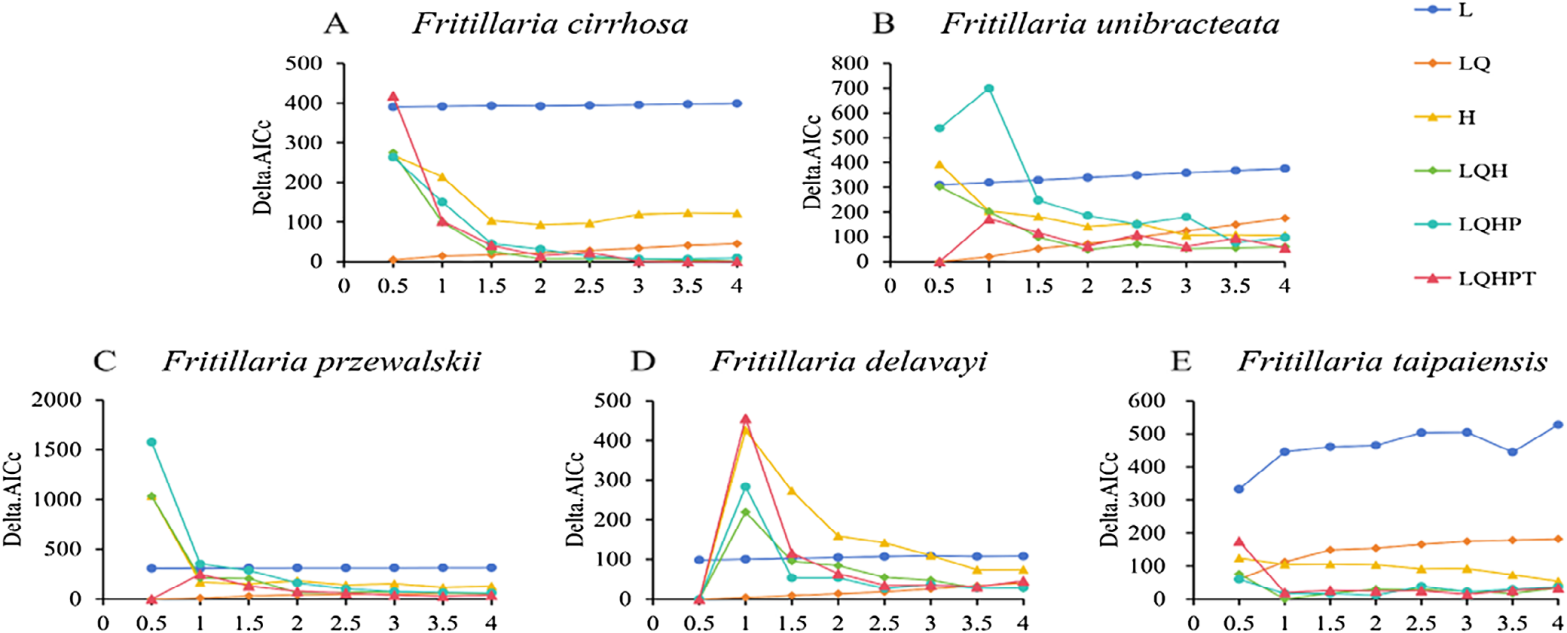
Variations in delta.AICc parameters in the optimized model. *F. cirrhosa* (a); *F. unibracteata* (b); *F. przewalskii* (c); *F. delavayi* (d); *F. taipaiensis* (e).

Based on delta.AICc = 0 (Figure 3), the optimal parameter combinations were selected for Maxent modeling. The final parameters chosen for the species were as follows: *F. cirrhosa* (FC = LQHPT, RM = 3.5), *F. unibracteata* (FC = LQ, RM = 0.5), *F. przewalskii* (FC = LQ, RM = 0.5), *F. delavayi* (FC = LQ, RM = 0.5), *F. taipaiensis* (FC = LQH, RM = 1). The ROC curve (Figure S2) assessed model accuracy, with average AUC values of *F. cirrhos*a (0.946), *F. unibracteata* (0.973), *F. przewalskii* (0.973), *F. delavayi* (0.968), and *F. taipaiensis* (0.975), confirming the high predictive accuracy of the Maxent model for all species.

### 3.3 Critical Environmental Factors

**FIGURE 4:**
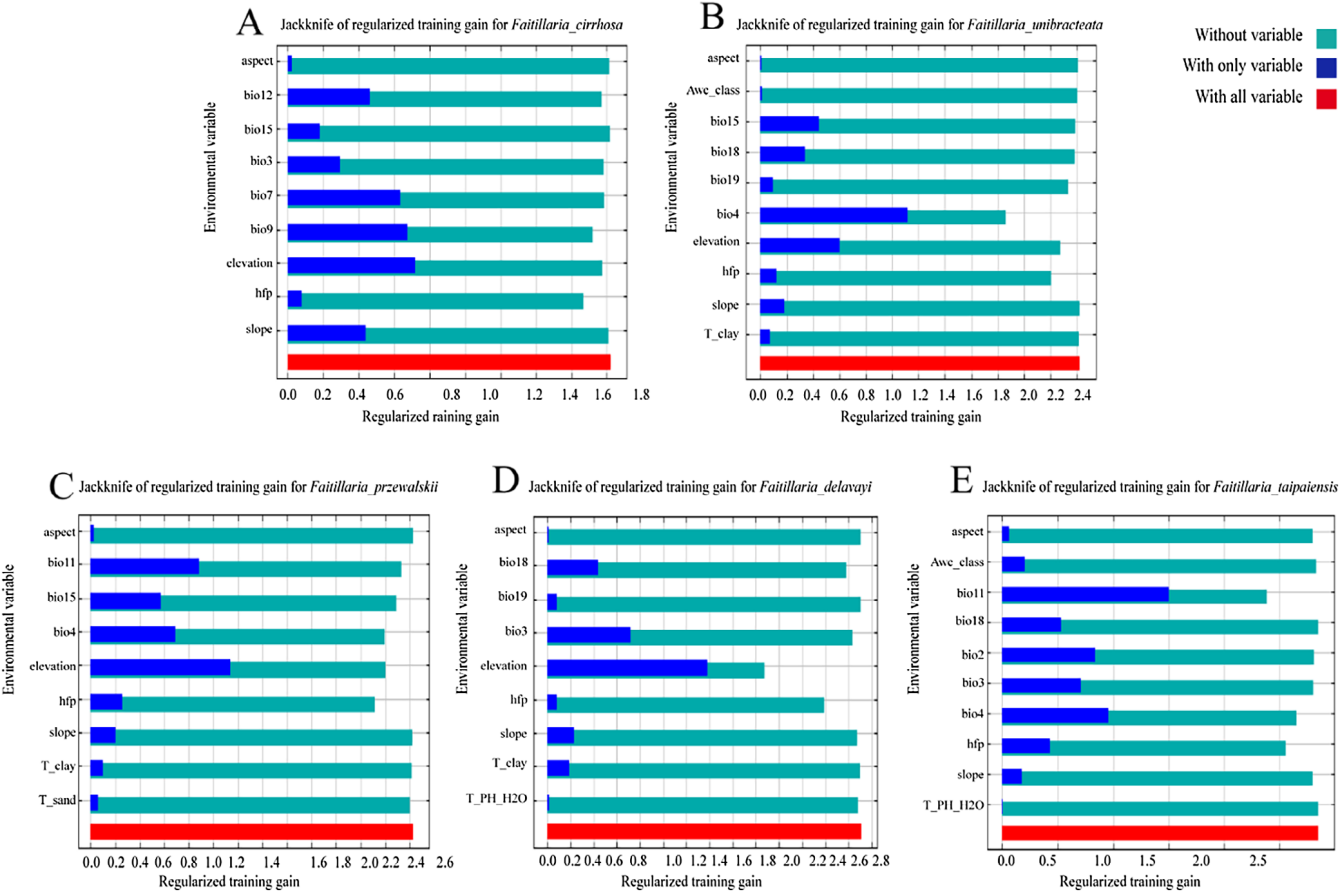
The results of the jackknife test of variable importance. *F. cirrhosa* (a); *F. unibracteata* (b); *F. przewalskii* (c); *F. delavayi* (d); *F. taipaiensis* (e).

This study identified the dominant environmental factors influencing the distribution of five *Fritillaria* species through contribution rates and jackknife test results (Yang 2024). As shown in Table S2 and Figure 4, the dominant environmental factors for each species were determined as follows: *F. cirrhosa*: bio7, bio9, bio12, elevation, and hfp, with a cumulative contribution of 95.8%; *F. unibracteata*: bio4, bio15, bio19, elevation, and hfp, with a cumulative contribution of 85.4%; *F. przewalskii*: bio4, bio11, bio15, elevation, and hfp, with a cumulative contribution of 98.1%; *F. delavayi*: bio3, bio18, elevation, and hfp, with a cumulative contribution of 93.8%; and *F. taipaiensis*: bio2, bio3, bio4, bio11, and hfp, with a cumulative contribution of 88.5%.

**FIGURE 5:**
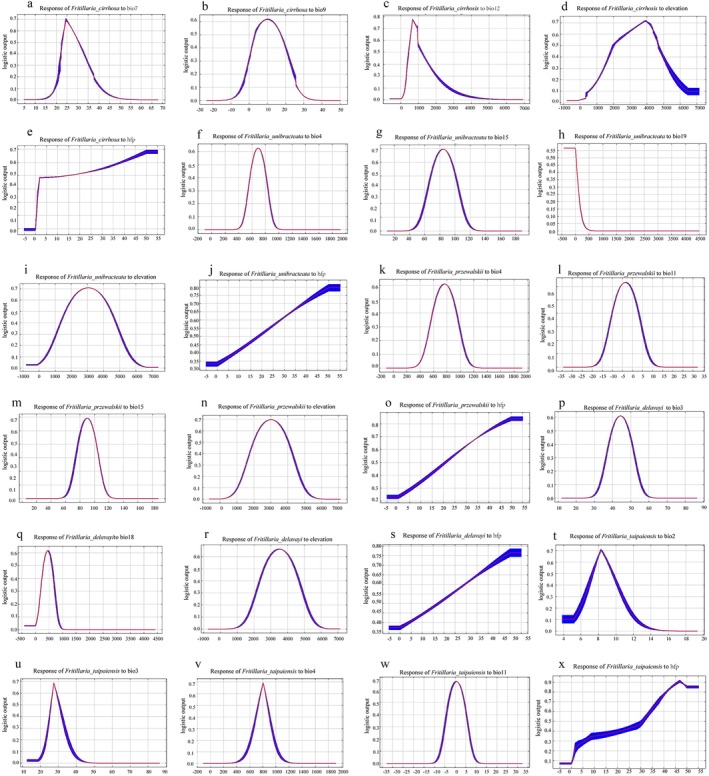
Response curves of some important environmental variables of five *Fritillaria* species. (a–e) bio7, bio9, bio12, elevation, and hfp of *F. cirrhosa*. (f–j) bio4, bio15, bio19, elevation, and hfp of *F. unibracteata*. (k–o) bio4, bio11, bio15, elevation, and hfp of *F*. *przewalskii*. (p–s) bio3, bio18, elevation, and hfp of *F. delavayi*. (t–x) bio2, bio3, bio4, bio11, and hfp of *F*. *taipaiensis*.

The response curves of environmental factors (Figure 5) reflect how suitability depends on specific variables. Probabilities greater than 0.5 indicate conditions suitable for the growth of five *Fritillaria* species (Li et al. 2025; Liu et al. 2025). For *F. cirrhosa*, optimal conditions were: bio7 (24°C–28°C, optimal at 25°C), bio9 (−3°C to 7.9°C, optimal at 0.1°C), bio12 (612–936 mm, optimal at 680 mm), and elevation (2751–4464 m, optimal at 3933 m). For *F. unibracteata*, the suitable ranges were: bio4 (648–734), bio15 (73–95), bio19 (≤ 23 mm), and elevation (2000–4092 m, optimal at 3046 m). For *F. przewalskii*, the suitable ranges were: bio4 (726–803), bio11 (−6°C to −1°C, optimal at −3°C), bio15 (81–98), and elevation (2274–3758 m, optimal at 3020 m). For *F. delavayi*, the suitable ranges were: bio3 (42–45), bio18 (399–505 mm, optimal at 457 mm), and elevation (2939–4085 m, optimal at 3500 m). For *F. taipaiensis*, the suitable ranges were: bio2 (7.7°C–9°C), bio3 (best at 26–29 mm), bio4 (767–838), and bio11 (−2°C–3°C, optimal at 0.6°C).

### 3.4 The Current Suitable Areas of Five *Fritillaria* Species Under Climate Change Scenarios

**FIGURE 6:**
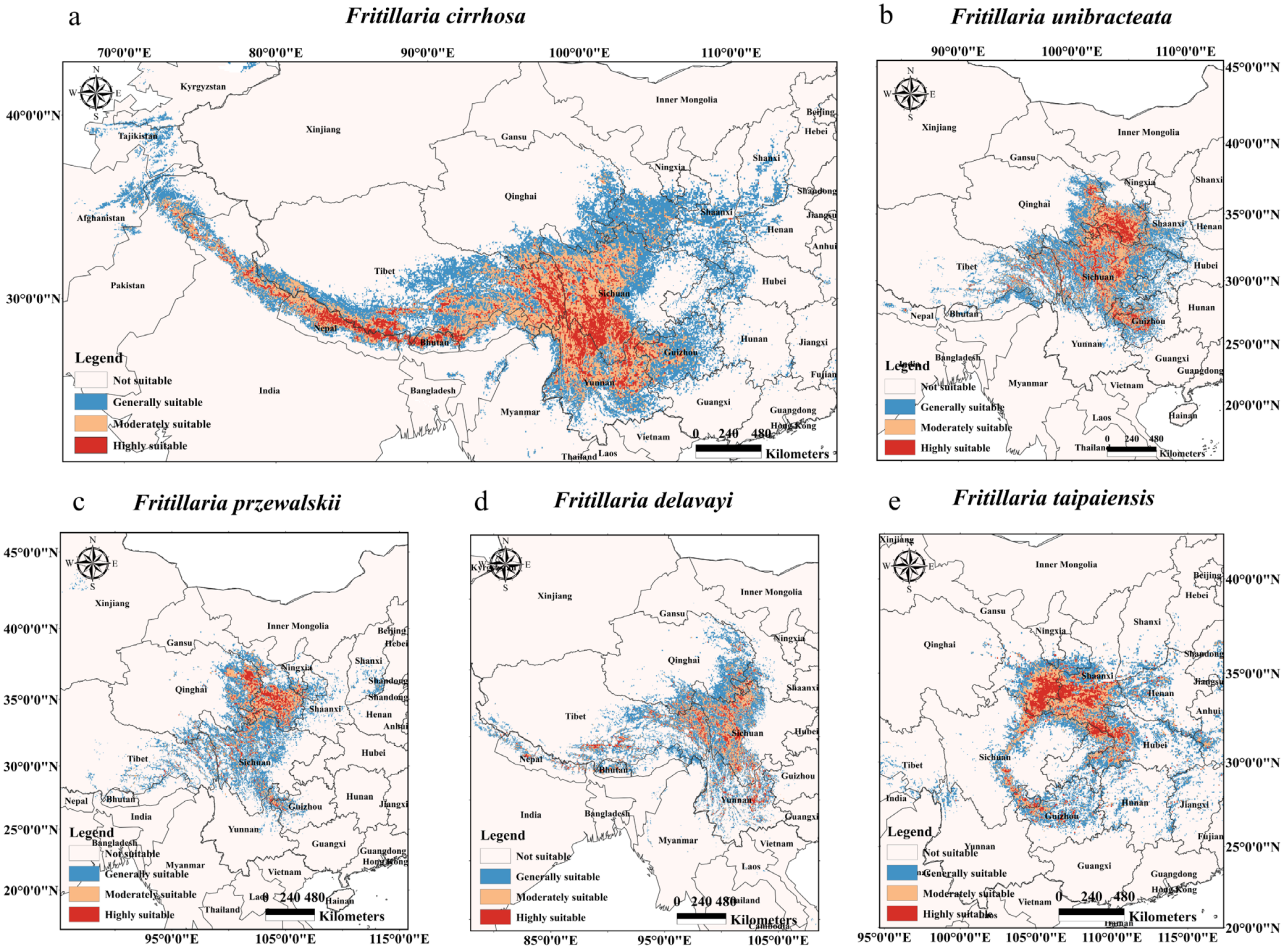
Distribution of suitable areas for five *Fritillaria* species under current climatic conditions. Distribution area (A); area (B); *F. cirrhosa* (a); *F. unibracteata* (b); *F. przewalskii* (c); *F. delavayi* (d); *F. taipaiensis* (e).

As shown in Table 2 and Figure 6, *F. cirrhosa* had a broad suitable range, while the other *Fritillaria* species had more concentrated distributions. *F. cirrhosa* was distributed from the northwestern Indian subcontinent, including Pakistan, to central eastern China, primarily along the southeastern Tibetan Plateau. It had areas with high suitability (1.36 × 10^4^ km^2^), moderately suitable (3.41 × 10^4^ km^2^), and generally suitable (6.28 × 10^4^ km^2^). *F. unibracteata* was found in northwestern Sichuan, southeastern Qinghai, and Tibet, with high suitability (0.73 × 10^4^ km^2^), moderately suitable (1.86 × 10^4^ km^2^), and generally suitable (3.23 × 10^4^ km^2^). *F. przewalskii* was distributed in southeastern Qinghai and southern Gansu, with high suitability (0.40 × 10^4^ km^2^), moderate suitability (1.07 × 10^4^ km^2^), and general suitability (2.77 × 10^4^ km^2^). *F. delavayi* was found in eastern Tibet, northwestern Yunnan, and western Sichuan, with high suitability (0.41 × 10^4^ km^2^), moderate suitability (1.30 × 10^4^ km^2^), and general suitability (2.85 × 10^4^ km^2^). *F. taipaiensis* was mainly distributed in southeastern Gansu, southern Shaanxi, northwestern Hubei, and Chongqing, with high suitability (0.79 × 10^4^ km^2^), moderate suitability (1.12 × 10^4^ km^2^), and general suitability (2.14 × 10^4^ km^2^).

**TABLE 2:** Changes in suitability areas and centroid for five *Fritillaria* species over time under different SSP scenarios.

Species	Scenario	Period	High suitability area (×10^−4^ km)	Moderate suitability area (×10^−4^ km)	General suitability area (×10^−4^ km)	Increase area (×10^−4^ km)	Lost area (×10^−4^ km)	Change area (×10^−4^ km)	Distance 1 (/km)	Distance 2 (/km)
*F. cirrhosae*		Current	1.36	3.41	6.28					
	SSP126	2041–2060	1.90	3.65	6.25	23.00	9.14	13.86	25.21	25.21
		2061–2080	2.71	3.34	5.24	20.01	15.07	4.94	42.01	61.55
	SSP245	2041–2060	2.49	3.66	6.14	31.14	9.36	21.78	62.24	62.24
		2061–2080	2.42	3.66	5.87	30.36	14.79	15.57	49.61	17.42
	SSP585	2041–2060	1.25	3.36	5.71	12.44	26.36	−13.93	37.72	37.72
		2061–2080	2.61	3.09	4.80	26.83	37.83	−11.00	56.33	45.00
*F. unibracteata*		Current	0.73	1.86	3.23					
	SSP126	2041–2060	1.35	1.97	2.94	10.97	3.01	7.96	16.70	16.70
		2061–2080	1.76	2.17	2.91	24.61	5.41	19.20	63.51	53.97
	SSP245	2041–2060	1.85	2.27	3.06	26.55	1.71	24.84	29.67	29.67
		2061–2080	1.45	2.06	3.18	18.79	2.70	16.09	32.98	35.67
	SSP585	2041–2060	1.70	2.22	3.18	26.02	2.09	23.94	69.51	69.51
		2061–2080	2.35	2.36	2.99	36.52	1.72	34.80	44.81	45.58
*F. przewalskii*		Current	0.40	1.07	2.77					
	SSP126	2041–2060	0.17	0.59	2.05	1.05	26.79	−25.75	80.08	80.08
		2061–2080	0.19	0.64	2.11	2.64	26.14	−23.50	73.97	37.07
	SSP245	2041–2060	0.29	0.94	2.48	6.51	16.18	−9.67	60.99	60.99
		2061–2080	0.18	0.67	2.37	2.03	20.41	−18.38	31.50	41.79
	SSP585	2041–2060	0.22	0.71	2.29	4.38	23.09	−18.71	81.17	81.17
		2061–2080	0.06	0.29	2.54	1.90	44.30	−42.40	147.93	186.47
*F. delavayi*		Current	0.41	1.30	2.85					
	SSP126	2041–2060	0.77	1.50	2.59	10.74	5.44	5.31	5.07	5.07
		2061–2080	0.99	1.75	2.45	18.00	6.34	11.66	13.18	8.12
	SSP245	2041–2060	1.33	1.80	2.53	24.32	4.73	19.58	51.75	51.75
		2061–2080	1.47	1.92	3.01	36.26	3.06	33.20	68.37	8.12
	SSP585	2041–2060	1.77	2.21	3.18	49.42	2.17	47.26	80.63	80.63
		2061–2080	1.80	1.97	2.73	38.86	3.44	35.42	49.27	34.42
*F. taipaiensis*		Current	0.79	1.12	2.14					
	SSP126	2041–2060	0.71	1.18	2.08	10.57	12.08	−1.51	63.02	63.02
		2061–2080	1.59	1.31	1.88	20.89	7.23	13.66	69.24	23.16
	SSP245	2041–2060	0.75	0.85	1.49	9.23	27.76	−18.52	181.54	181.54
		2061–2080	0.35	0.72	1.46	5.13	33.62	−28.49	206.84	36.47
	SSP585	2041–2060	0.43	0.78	1.57	7.18	31.39	−24.21	267.32	267.32
		2061–2080	0.54	0.84	1.37	9.41	34.15	−24.74	248.08	20.25

*Note:* Distance 1 is the distance from the current centroid. Distance 2 is the distance from the centroid of the previous period.

### 3.5 The Future Suitable Areas of Five *Fritillaria* Species Under Climate Change Scenarios

Studies indicate that over 80% of species' distributions are influenced by climate change, with migration patterns strongly linked to warming. Table 2 showed the area variations and changing trends of the potentially suitable habitats for five *Fritillaria* species under the SSP126, SSP245, and SSP585 scenarios spanning from 2041 to 2060 and 2061 to 2080. During these distinct SSP scenarios, differences emerged in the suitable area alterations for each original *Fritillaria* species.

Under the SSP126 scenario: The high suitability, moderate suitability, and general suitability areas of *F. cirrhosa* exhibited an upward trend, a pattern of first rising then falling, and a downward trend respectively. The variety for *F. delavayi* showed increasing trends, while for *F. taipaiensis*, they displayed a sequence of first increasing then decreasing, increasing, and decreasing trends.

When it comes to the SSP245 scenario, the high suitability, moderate suitability, and general suitability areas of *F. cirrhosa* followed decreasing, increasing, and decreasing trends respectively. In contrast, *F. delavayi*'s corresponding areas demonstrate increasing, increasing, and a trend of first declining and then rising.

Under the SSP585 scenario, the change of *F. cirrhosa* presented a pattern of first decreasing then increasing, a continuous decrease, and further decline respectively. The variety of *F. delavayi* showed increasing, increasing, and a tendency of first rising then falling in these areas. Regarding *F. unibracteata*, across the SSP126, SSP245, and SSP585 scenarios, both the high and moderate suitability areas were on the rise, while the generally suitable area was shrinking. For *F. przewalskii*, all suitable areas showed a downward trend in every scenario. As for *F. taipaiensis*, under the SSP245 and SSP585 scenarios, its high and moderate suitability areas experienced increasing and decreasing trends respectively. Specifically, during the period from 2061 to 2080 under the SSP126 scenario, the area of the high suitability zone for *F. cirrhosa* hit a peak of 2.71 × 10^4^ km. Similarly, in the 2061 to 2080 SSP585 scenario, the high suitability area of *F. unibracteata* reached its maximum at 2.35 × 10^4^ km, and under the SSP126 scenario in the same period, the high suitability area of *F. delavayi* peaked at 1.59 × 10^4^ km.

**FIGURE 7:**
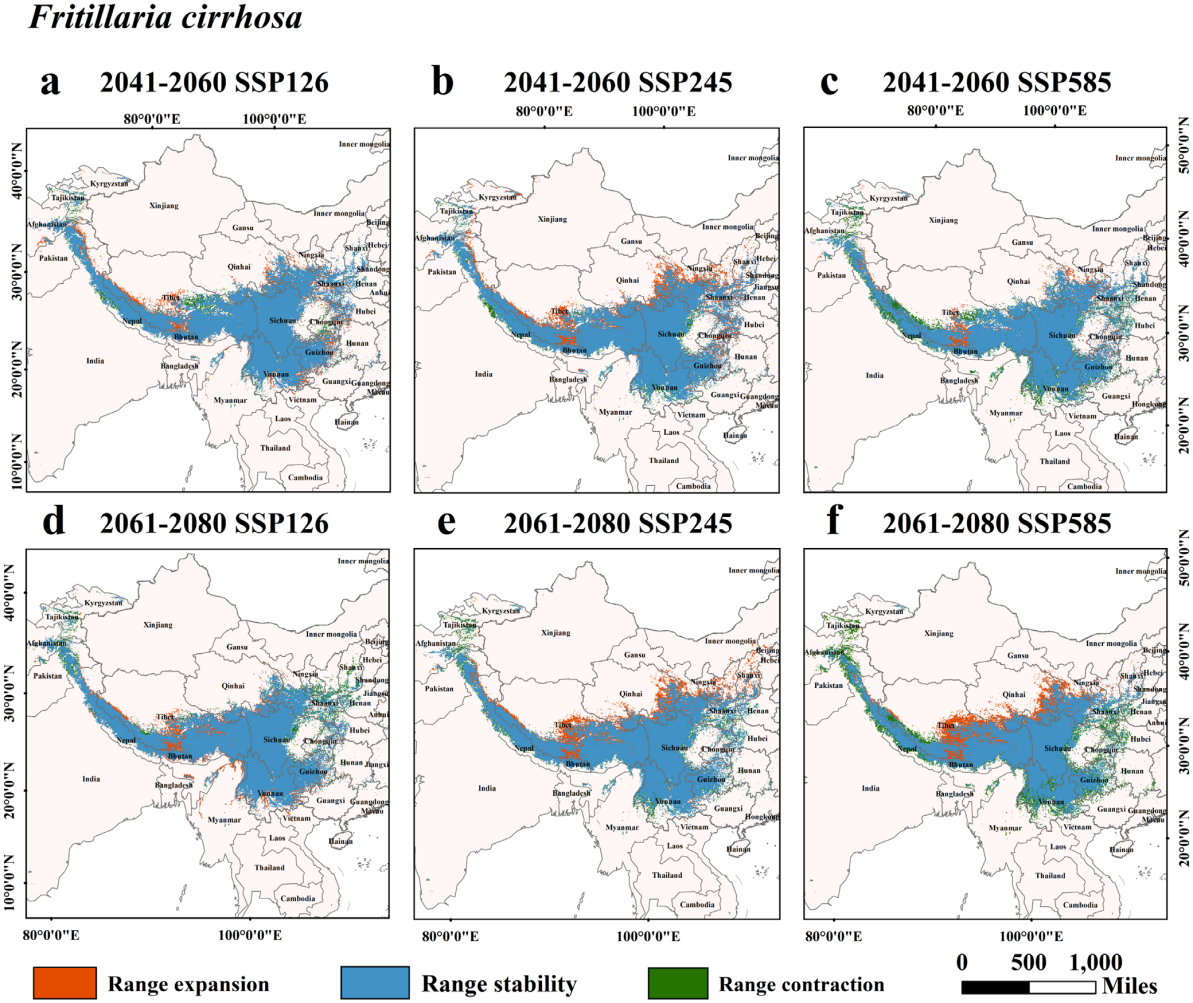
Changes in the distribution of suitable habitat for *F. cirrhosa* under future climate conditions. (a) SSP126 (2041–2060), (b) SSP245 (2041–2060), (c) SSP585 (2041–2060), (d) SSP126 (2061–2080), (e) SSP245 (2061–2080), (f) SSP585 (2061–2080).

**FIGURE 8:**
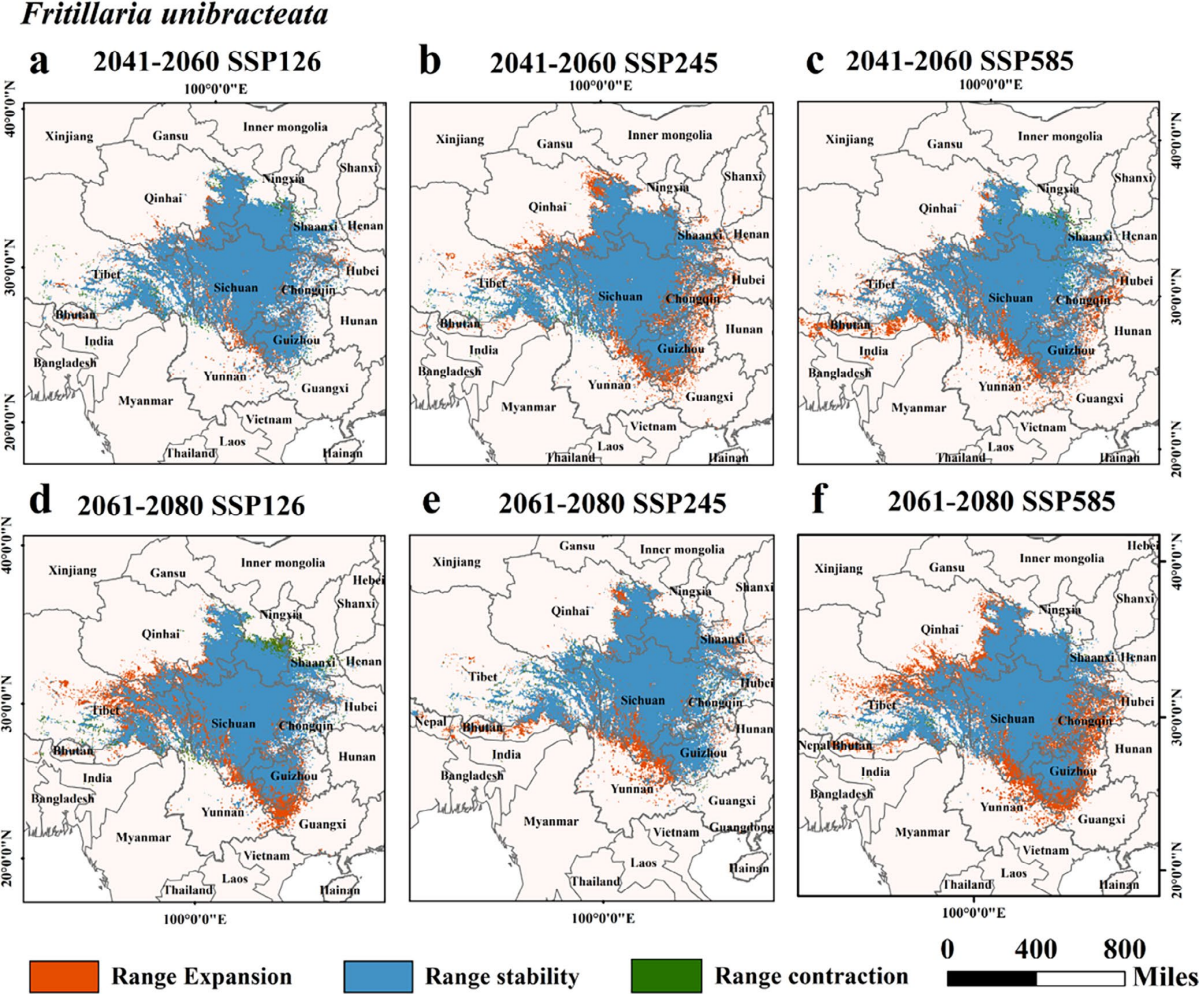
Changes in the distribution of suitable habitat for *F. unibracteata* under future climate conditions. (a) SSP126 (2041–2060), (b) SSP245 (2041–2060), (c) SSP585 (2041–2060), (d) SSP126 (2061–2080), (e) SSP245 (2061–2080), (f) SSP585 (2061–2080).

**FIGURE 9:**
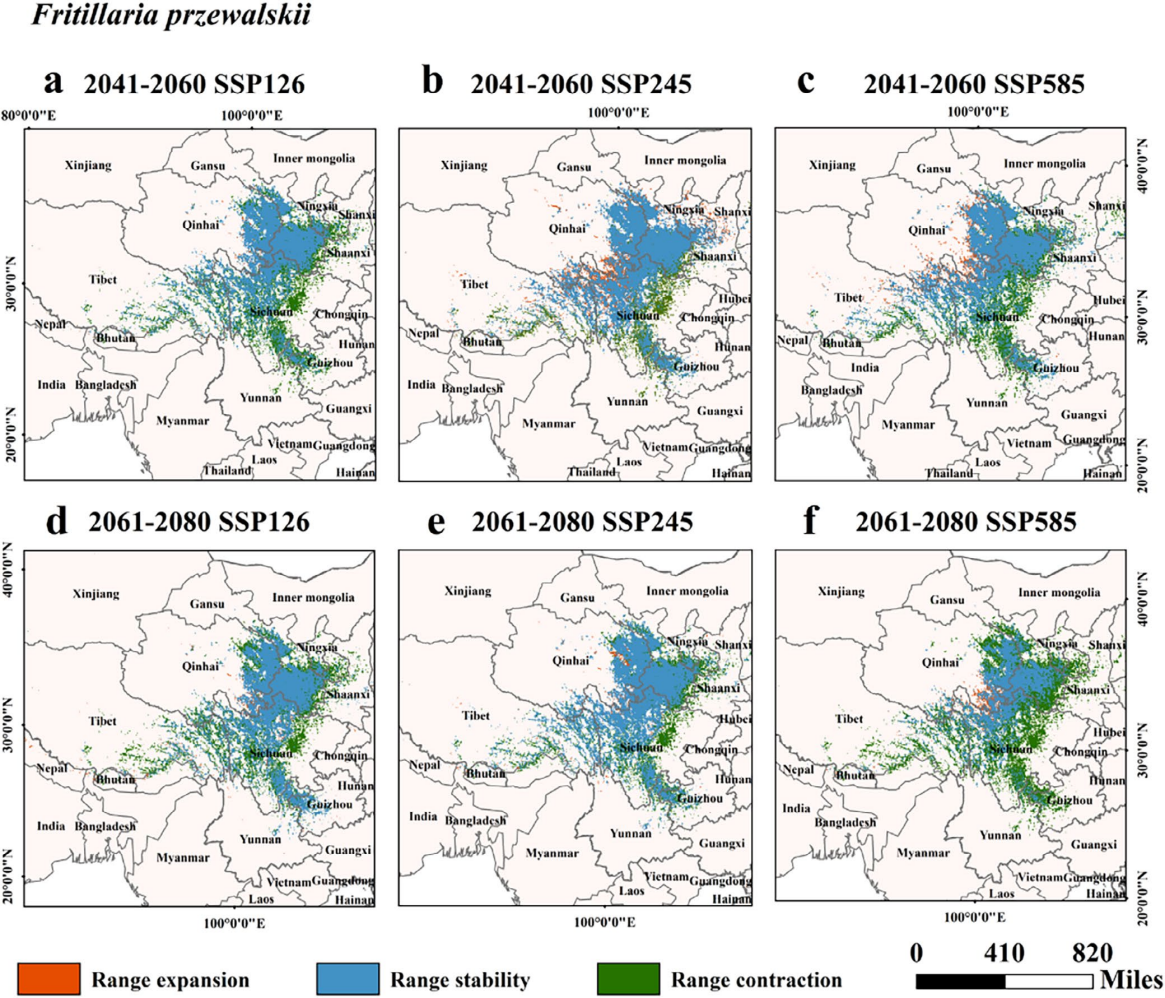
Changes in the distribution of suitable habitat for *F. przewalskii* under future climate conditions. (a) SSP126 (2041–2060), (b) SSP245 (2041–2060), (c) SSP585 (2041–2060), (d) SSP126 (2061–2080), (e) SSP245 (2061–2080), (f) SSP585 (2061–2080).

**FIGURE 10:**
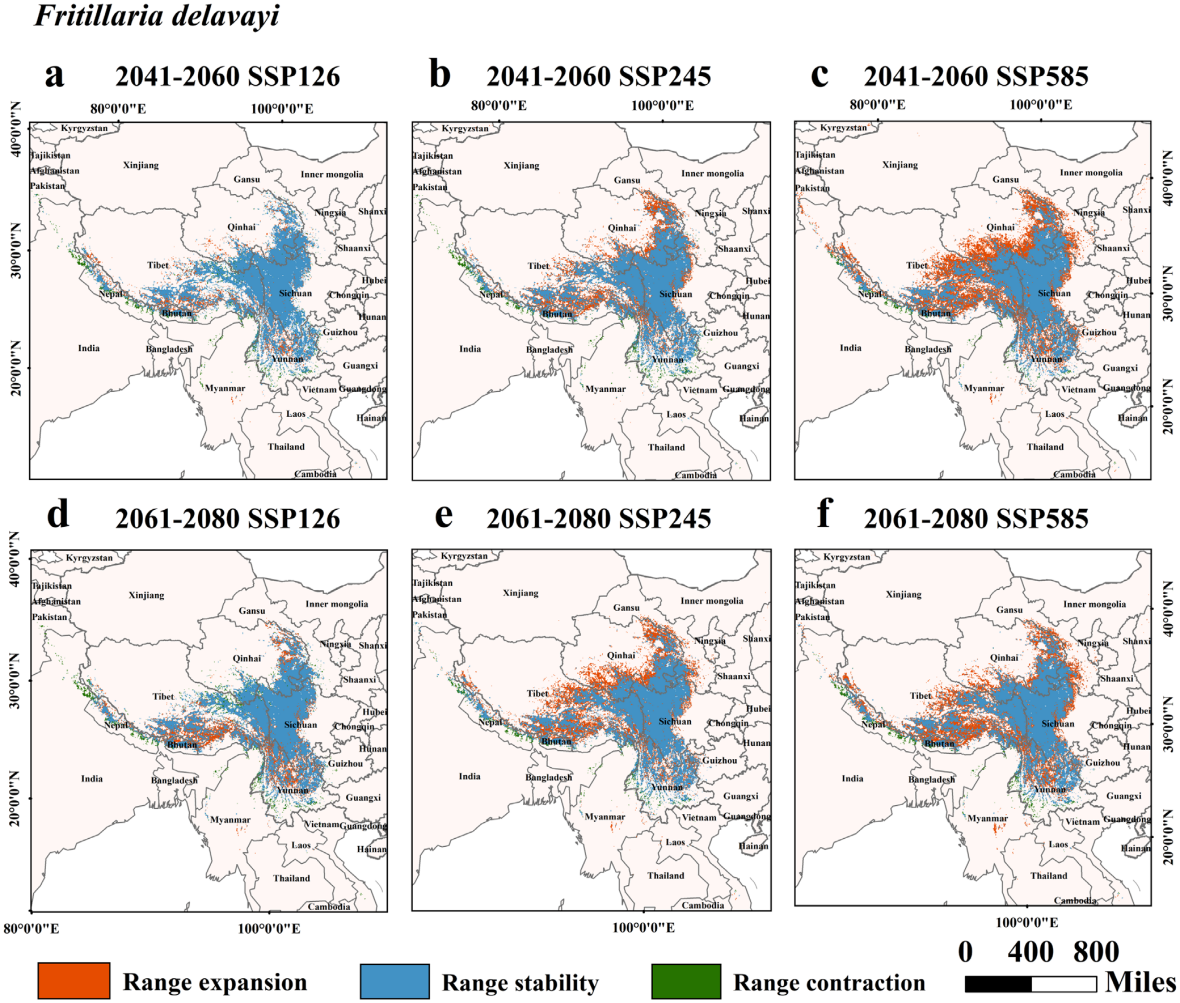
Changes in the distribution of suitable habitat for *F. delavayi* under future climate conditions. (a) SSP126 (2041–2060), (b) SSP245 (2041–2060), (c) SSP585 (2041–2060), (d) SSP126 (2061–2080), (e) SSP245 (2061–2080), (f) SSP585 (2061–2080).

**FIGURE 11:**
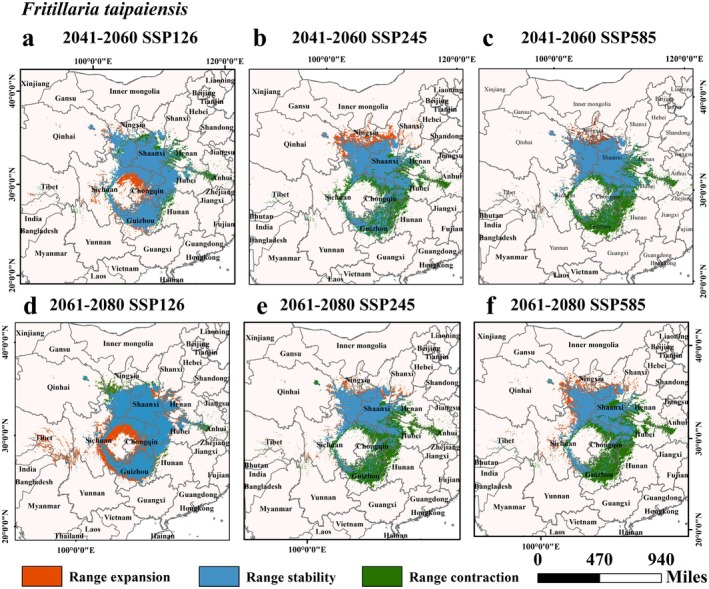
Changes in the distribution of suitable habitat for *F. taipaiensis* under future climate conditions. (a) SSP126 (2041–2060), (b) SSP245 (2041–2060), (c) SSP585 (2041–2060), (d) SSP126 (2061–2080), (e) SSP245 (2061–2080), (f) SSP585 (2061–2080).

Data from Table 2 and Figures 7, 8, 9, 10, 11 suggested that under future climate conditions, the total suitable areas of *F. unibracteata* and *F. delavayi* were expanding, while those of *F. przewalskii* and *F. taipaiensis* were contracting. Moreover, the total suitable area of *F. cirrhosa* grew under the SSP126 and SSP245 scenarios but shrank under the SSP585 scenario.

### 3.6 Centroid Shifts of Five *Fritillaria* Species Under Climate Change Scenarios

**FIGURE 12:**
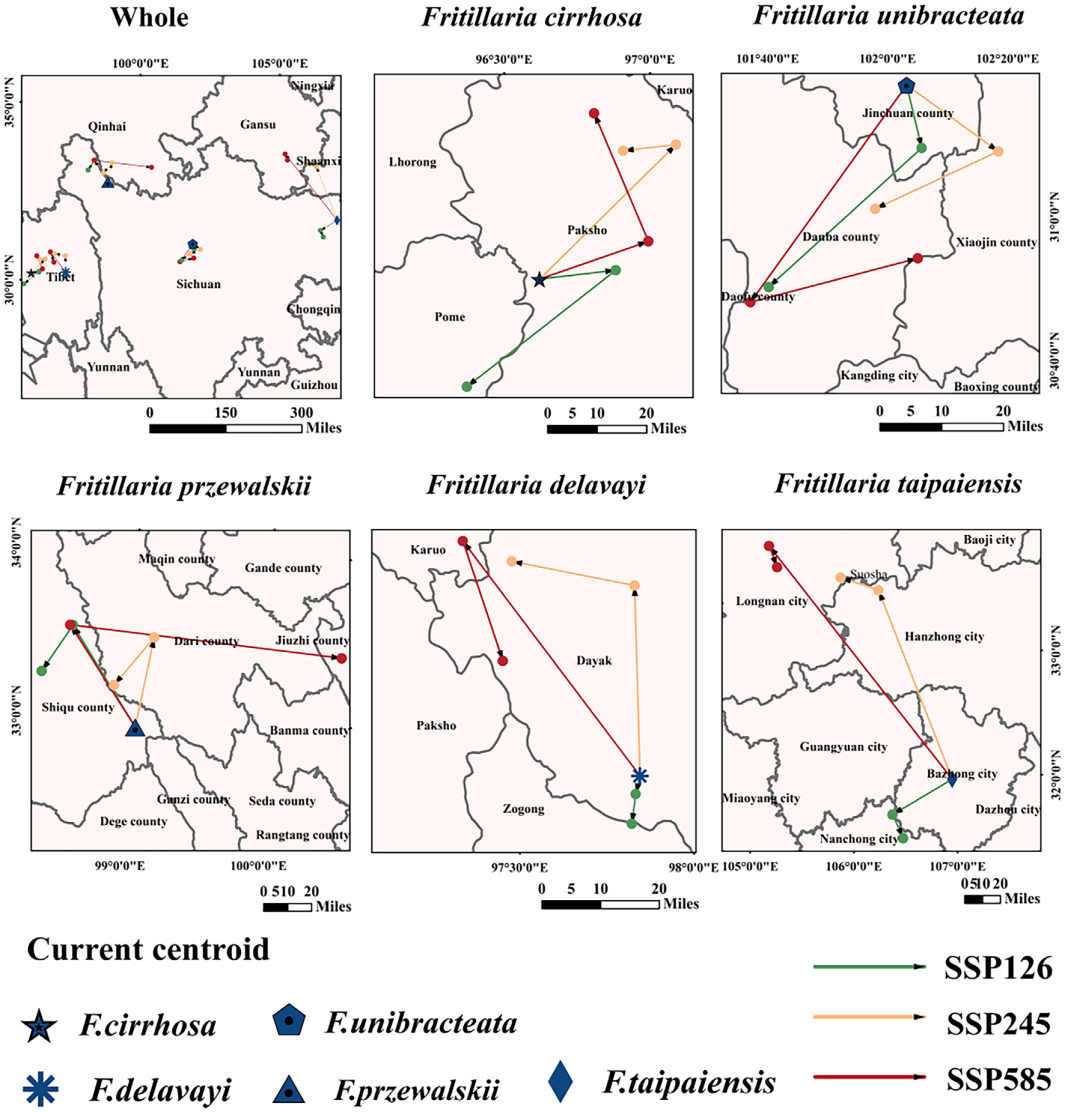
Geographical distribution changes in the centroid of the suitable growing area of five *Fritillaria* species under different climate change scenarios.

The trajectory of centroid shifts for five *Fritillaria* species' suitable habitats was shown in Figure 12 and Table 2. For *F. cirrhosa*, under SSP126, the centroid shifted east from 2041 to 2060, then southwest from 2061 to 2080. In both the SSP245 and SSP585 scenarios, the centroid shifted northeast during both periods. For *F. unibracteata*, under SSP126 and SSP245, the centroid shifted southeast from 2041 to 2060, then southwest from 2061 to 2080. In SSP585, it shifted southwest during 2041–2060, then south in 2061–2080. For *F. przewalskii*, under SSP126, the centroid shifted northwest in both periods. Under SSP245, it shifted northeast from 2041 to 2060, then backed to northwest from 2061 to 2080. In SSP585, the centroid remained northwest during 2041–2060, then shifted northeast in 2061–2080. For *F. delavayi* and *F. taipaiensis*, under SSP126, the centroids shifted south in both periods. Under SSP245, they shifted north, while in SSP585, they shifted northwest.

## 4 Discussion

### 4.1 Differences in Quantity of Specimens and Correlation Between the Elevation Distribution and Time of Five *Fritillaria* Species

Over the past nearly 70 years, China has experienced significant climate changes that have greatly influenced the elevation distribution of various species (Li et al. 2019; Zu 2021). In the context of global warming, most species are showing an upward‐shifting trend in their elevation ranges. However, some species are either shifting downward or not shifting at all (Lenoir et al. 2010). Our research indicates that the resource distributions of *F. przewalskii* and *F. delavayi* are migrating to higher elevations over time, which is consistent with the observations of many other plant species. For example, *Paeonia delavayi* , *Paeonia rockii* (Zhang et al. 2018), *Satyrium nepalense* (Shen et al. 2023), and *Akebia trifoliata* (Jm et al. 2022) are also shifting to higher latitudes or elevations. In contrast, the resource distribution of *F. taipaiensis* is moving toward lower elevations. Our findings demonstrate that *F. taipaiensis* exhibits predominantly lower elevations compared to its congeners. This variety is most suitable for cultivation at low elevations (Qian 2018). Our research findings, along with previous studies, indicate that species in higher mountain areas tend to shift to higher elevations, while those in lower areas shift downward (Zu et al. 2023). For example, 14 species of *Rhodiola* have been observed to shift upward at a rate of 0.27–7.07 m per year, likely due to rising global temperatures since the Last Glacial Maximum (You et al. 2018). Additionally, the elevations of *Ranunculus tanguticus* (Maxim.) Ovcz. and *Rhododendron nitidulum* Rehd. et Wils have decreased by 190 and 145 m, respectively, influenced by changes in precipitation (Zu et al. 2023). Similarly, *Paederia scandens* and *Pericampylus glaucus*, which are distributed in lowland areas, have also shifted downward by 177.4 and 94.9 m, respectively, due to these precipitation changes (Zu et al. 2023). Our study showed that the resource distribution of *F. cirrhosa* and *F. unibracteata* did not exhibit a discernible trend of change. It has been pointed out that widely distributed species tend to have greater environmental adaptability and are less affected by external changes (Holt 2003). We found that the quantities of these two *Fritillaria* species were relatively large and their distributions were extensive. This might explain why their resource distributions have not shown a specific trend of change.

### 4.2 Differences in Five *Fritillaria* Species Affected by Dominant Environmental Factors

Dominant environmental drivers of habitat suitability exhibit significant interspecific variation (Fang et al. 2023). For example, bio9 critically determines the distribution of *Acer tegmentosum* and *Acer pseudo‐sieboldianum* (Kabaš et al. 2014), while bio11 and bio18 governed the range limits of *Nardostachys jatamansi* (Wang and He 2019). Our analyses further demonstrated that the five *Fritillaria* species responded to distinct suites of environmental factors, reflecting species‐specific ecological strategies. These factors play a crucial role in shaping species distribution and survival, which in turn influence the structure of the plant community. According to the response surface curves, it is recommended to plant *F. cirrhosa* and *F. delavayi* at elevations between approximately 3500 and 3900 m. For *F. unibracteata* and *F. przewalskii*, planting is best done at around 3000 m above sea level. Additionally, it is more beneficial for the growth of *F. cirrhosa* to maintain the temperature of its planting environment at around 25°C.

Among the five *Fritillaria* species studied, four are influenced by elevation, which aligns with previous studies by Jiang et al. (2016) and Zhao, Chen, et al. (2018), identifying elevation as a primary factor affecting the distribution of five *Fritillaria* species. Elevation plays a crucial ecological role in biological growth, impacting various ecological parameters such as temperature, precipitation, and soil conditions, which in turn influence the species distributions (Körner 2007; Li et al. 2020). Five *Fritillaria* species exhibit poor growth and reproductive performance at low elevations, with both bulb and total plant biomass declining at higher elevations (Chen et al. 2012). Meanwhile, elevation affects leaf area, plant height, and specific leaf area (Xu et al. 2014). The results from the response surface curve indicate that the suitable elevation range for the species is as follows: *F. cirrhosa* (2751–4464 m), *F. unibracteata* (2000–4092 m), *F. przewalskii* (2274–3758 m). The optimal range for *F. delavayi* is between 2959 and 4085 m. These findings suggest that *F. unibracteata* has the widest adaptation to elevation. These results underscore significant differences in elevation adaptation among the species, despite their shared classification within the *Fritillaria* species. These differences are likely influenced by their respective physiological traits, which have developed throughout their evolutionary histories, shaping their distribution patterns and resource utilization strategies.

### 4.3 Differences in the Distribution of Potential Areas of Five *Fritillaria* Species Under Climate Change Scenarios

Global environments are undergoing changes as atmospheric carbon dioxide (CO_2_) concentrations continue to rise, profoundly impacting plant growth (Morison and Lawlor 1999). Wild plants exhibit diverse adaptive behaviors in response to climate change, causing species' suitable distribution areas to expand or contract under various future climate scenarios (Liang et al. 2018). In this study, we found that under high greenhouse gas emission scenarios (SSP585), the total suitable habitat area for *F. unibracteata* and *F. przewalskii* increases. This finding is consistent with the results of Liu et al. (2021) and Zhou (2022). Moreover, it aligns with the Maxent model predictions for other species, such as *Cynaeus angustus* (Zhao et al. 2023) and *Cunninghamia lanceolata* (Zhao et al. 2021), both of which also indicate an expanding trend in their overall suitable habitats. Moderate temperature increases may benefit the reproduction of these plants or their host species. Xu et al. (2019) underscore that species respond differently to human activities, with distinct patterns between widely distributed species and those with restricted ranges. Specifically, narrowly distributed species are more negatively affected by human activities. Consistent with this, our study found that the total suitable habitat areas for *F. przewalskii* and *F. taipaiensis* decreased under the SSP585 scenario. This reduction is likely due to increased human activities intensity—including factors such as urban development, population density, farmland, pastures, roads, and railways—which diminishes the survival probabilities of these species and fragments their distribution areas. These findings suggest that these species are highly sensitive to human‐induced disturbances.

However, our results contradict the trends reported by Zhao, Li, et al. (2018). This inconsistency may arise from methodological differences, as it adopted different environmental factors from ours and used alternative climate scenario models, such as CCCMA, CSIRO, and HCCPR, which differ from those employed in our study. These variations in model parameters and climate data selection likely account for the disparities in the predictions.

To expand suitable habitats for *Fritillaria* species, it is essential to focus monitoring and protection efforts on new regions. Carefully planned measures should be implemented to ensure population stability. For *F. taipaiensis*, which is experiencing a reduction in its suitable habitat, it is critical to monitor existing habitats and implement targeted protection efforts. This includes safeguarding surrounding ecosystems to prevent further decline. Additionally, resource development should be aligned with species' distribution trends, using scientifically informed plans to prevent over‐collection and population harm.

### 4.4 Limitations of the Study

This study elucidates the spatiotemporal dynamics of five *Fritillaria* species' potential distribution under climate change, offering a scientific basis for conservation strategies. However, limitations include: simplified human activity dynamics, as future projections assume the static human footprint owing to data constraints, neglecting potential interactions between evolving anthropogenic pressures and climate change. Insufficient incorporation of biotic interactions, as predictions rely mainly on abiotic factors (climate, topography) without key ecological processes such as competition, predation, or symbiosis (Higgins et al. 2003; Liu et al. 2022). These biotic factors significantly influence the distribution patterns of species. To consider these factors, it will be necessary to build a more complex and integrated SDMs for simulation. Nevertheless, the optimized Maxent model achieved high predictive accuracy (AUC > 0.9), ensuring reliable results. Thus, this study remains valuable for understanding five *Fritillaria* species' climate response and guiding its conservation and sustainable management.

## 5 Conclusion

The study revealed that the elevation, bio4, and hfp are the dominant environmental drivers governing the distribution patterns of the five *Fritillaria* species. *F. przewalskii* and *F. delavayi* have migrated to higher elevations, while *F*. *taipaiensis* has moved to lower elevations. In contrast, *F. cirrhosa* and *F. unibracteata* have extensive suitable habitats and demonstrate high adaptability, showing no signs of migration. Under the highest greenhouse gas emissions scenario, the suitable habitats for these species are expected to change. The total suitable areas of *F. cirrhosa*, *F. przewalskii*, and *F. taipaiensis* are projected to shrink, whereas the habitats for *F. unibracteata* and *F. delavayi* will likely expand. Additionally, their geographical centroids are expected to shift. To ensure the long‐term survival and reproduction of these species, it is essential to develop strategies for wild tending and artificial cultivation that take into account each species' environmental requirements and the anticipated impacts of climate changes.

## Author Contributions

**Yuanyuan Li:** data curation (equal), formal analysis (equal), investigation (equal), visualization (equal), writing – original draft (equal). **Qinghe Wang:** conceptualization (equal), supervision (equal), writing – review and editing (equal). **Rong Ding:** methodology (equal), writing – review and editing (equal). **Xiaofen Liu:** investigation (equal), writing – review and editing (equal). **Sijing Liu:** writing – review and editing (equal). **Jing Bai:** writing – review and editing (equal). **Shuqi Niu:** writing – review and editing (equal). **Jinlin Guo:** funding acquisition (equal), project administration (equal), resources (equal), writing – review and editing (equal).

## Conflicts of Interest

The authors declare no conflicts of interest.

## Supporting information


**Appendix S1:** ece372305‐sup‐0001‐AppendixS1.docx.

## Data Availability

Raw distribution data, environment variables, and R code are available in figshare: https://figshare.com/articles/dataset/__R_/28377875.
