# Supplementary material for: Comprehensive Analysis of the Impact of Climate Change and Human Activities on the Distribution of Five Fritillaria Species Using the Optimized Maxent Model
Source: Ecol Evol. 2025 Oct 13;15(10):e72305. doi: 10.1002/ece3.72305 (PMC12518166; doi:10.1002/ece3.72305)
Supplement: Supplementary file 1 — Appendix S1: ece372305‐sup‐0001‐AppendixS1.docx. [file ECE3-15-e72305-s001.docx]

**Supplementary Material**

**Comprehensive analysis of the impact of climate change and human activities on the distribution of five *Fritillaria* species using the optimized Maxent model**

Yuanyuan Li^a1^, Qing-he Wang^a1^, Rong Ding^b^, Xiaofen Liu^a^, Sijing Liu^c^, Jing Bai^c^, Shuqi Niu^c^, Jinlin Guo^a, b*^

^a^ State Key Laboratory of Southwestern Chinese Medicine Resources, College of Pharmacy, Chengdu University of Traditional Chinese Medicine, Chengdu 611137, China.

^b^ School of Ethnic Medicine, Chengdu University of Traditional Chinese Medicine, Chengdu 611137, China.

^c^ College of Medical Technology, Chengdu University of Traditional Chinese Medicine, Chengdu 611137, China.

^*^ Correspondence: JinLin Guo.

Email address: [guo596@cdutcm.edu.cn](mailto:guo596@cdutcm.edu.cn).

1. These authors’contributed equally.

**Table S1** Initial environment variable.

| Abbreviation | Climate variables | Unit |
| --- | --- | --- |
| Bio1 | Annual mean temperature | ℃ |
| Bio2 | Mean diurnal range | ℃ |
| Bio3 | Isothermality (bio2 / bio7) (× 100) | - |
| Bio4 | Temperature Seasonalit (standard deviation×100) | - |
| Bio5 | Max temperature of warmest month | ℃ |
| Bio6 | Min temperature of coldest month | ℃ |
| Bio7 | Temperature annual range (bio5- bio6) | ℃ |
| Bio8 | Mean temperature of wettest quarter | ℃ |
| Bio9 | Mean temperature of driest quarter | ℃ |
| Bio10 | Mean temperature of warmest quarter | ℃ |
| Bio11 | Mean temperature of coldest quarter | ℃ |
| Bio12 | Annual precipitation | mm |
| Bio13 | Precipitation of wettest month | mm |
| Bio14 | Precipitation of driest month | mm |
| Bio15 | Precipitation seasonality (Coefficient of variation) | - |
| Bio16 | Precipitation of wettest quarter | mm |
| Bio17 | Precipitation of driest quarter | mm |
| Bio18 | Precipitation of warmest quarter | mm |
| Bio19 | Precipitation of coldest quarter | mm |
| Elev | Altitude | m |
| Slope | Slope | ° |
| Aspect | Aspect | rad |
| Awc_class | Awc range | code |
| T_sand | Sand soil content | % |
| T_oc | Topsoil organic carbon | % weight |
| T_ph_h2o | Topsoil pH (H_2_O) | -log (H^+^) |
| T_clay | Topsoil clay fraction | % wt. |
| Hfp | Human foot print | - |

**
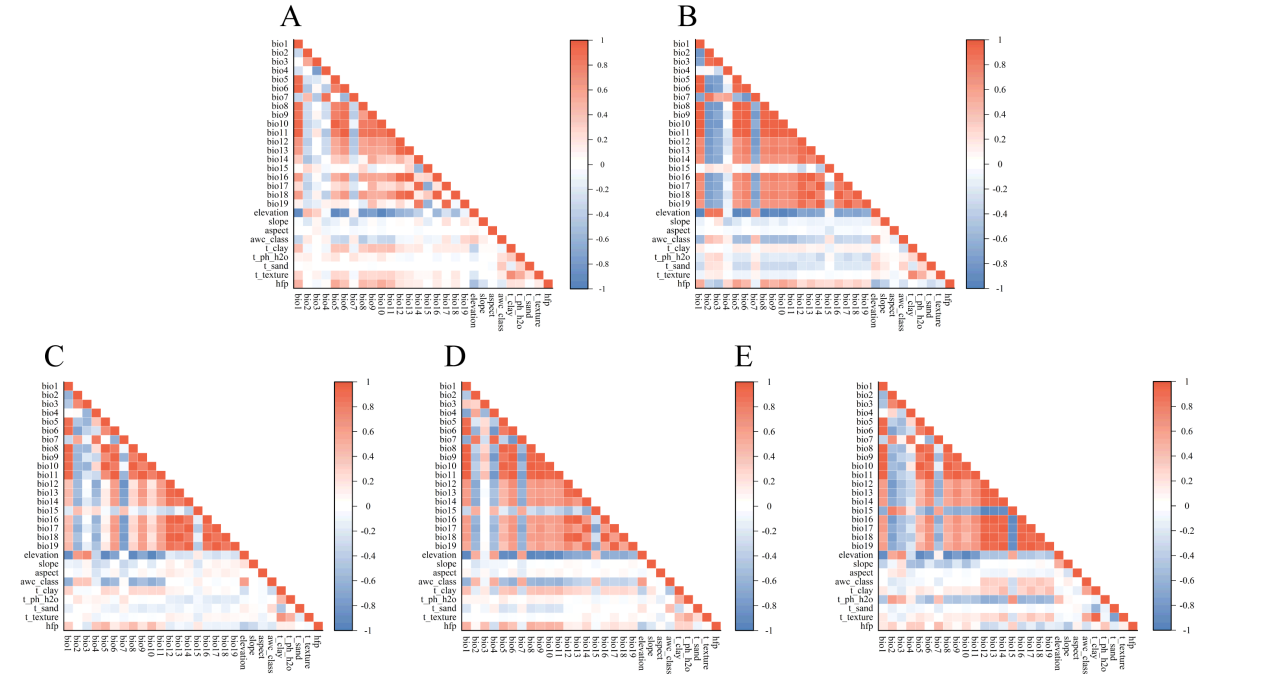
**

**Figure S1** Correlation coefficient matrix of environment variables. *F. cirrhosa* (A); *F. unibracteata* (B); *F. przewalskii* (C); *F. delavayi* (D); *F. taipaiensis* (E)*.*

***
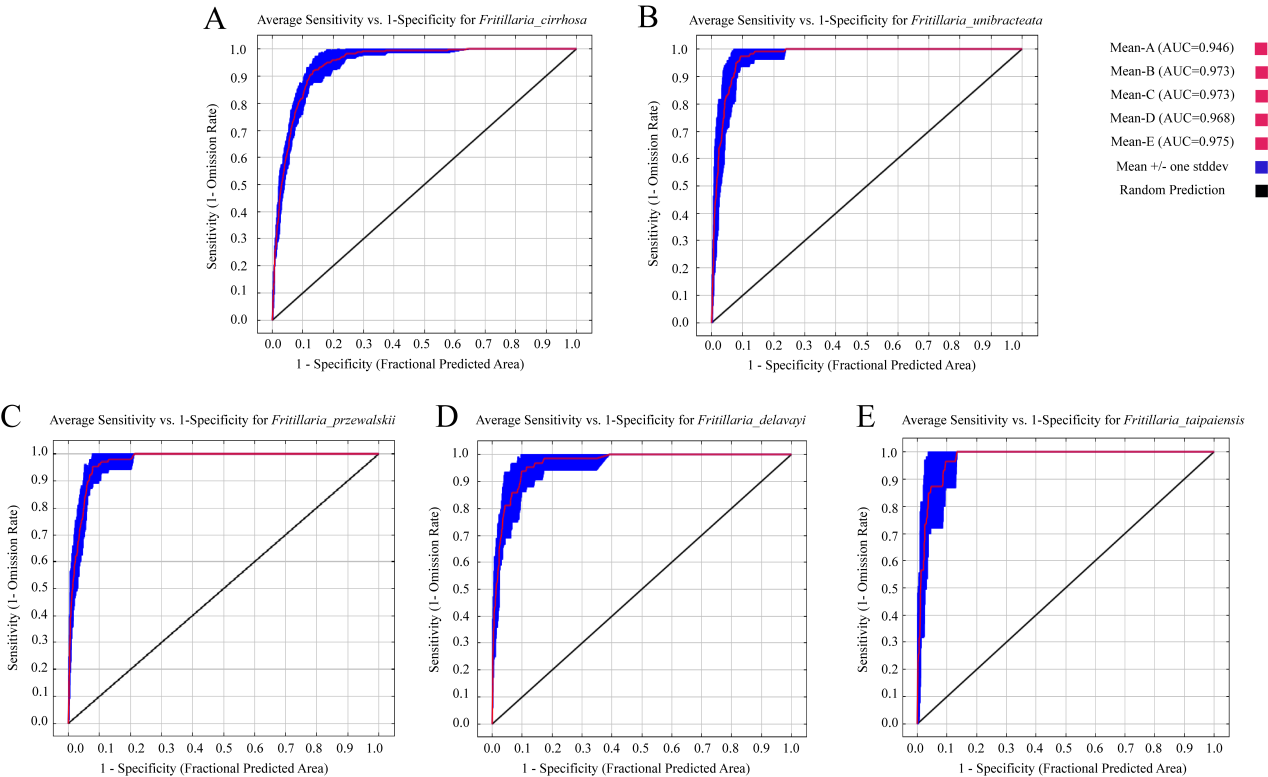
***

**Figure S2** The five *F.* species’ ROC curve and the values of AUC for the Maxent model. *F. cirrhosa* (A); *F. unibracteata* (B); *F. przewalskii* (C)*; F. delavayi* (D); *F. taipaiensis* (E)*.*

**Table S2** The contribution rate of various environmental factors.

| Species | Variable | Percent contribution (%) | Permutation importance (%) | Species | Variable | Percent contribution (%) | Permutation importance (%) |
| --- | --- | --- | --- | --- | --- | --- | --- |
| *F. cirrhosa* | Elevation | 35.3 | 17.1 | *F. przewalskii* | Elevation | 35.8 | 7.8 |
|  | Bio9 | 20.7 | 46.5 |  | Bio11 | 32.6 | 37 |
|  | Bio7 | 19.5 | 7.9 |  | Hfp | 19.9 | 5.1 |
|  | Bio12 | 11.7 | 14.3 |  | Bio4 | 6.7 | 40.6 |
|  | Hfp | 8.6 | 6.7 |  | Bio15 | 3.1 | 8.8 |
|  | Bio3 | 2.6 | 6.2 |  | T_sand | 0.7 | 0.4 |
|  | Slope | 1 | 0.4 |  | T_clay | 0.6 | 0.3 |
|  | Aspect | 0.5 | 0.6 |  | Slope | 0.3 | 0 |
|  | Bio15 | 0.2 | 0.4 |  | Aspect | 0.3 | 0 |
| *F.* *unibracteata* | Elevation | 29.4 | 6.2 | *F. delavayi* | Bio11 | 46.1 | 48.2 |
|  | Bio4 | 24.8 | 73.4 |  | Bio2 | 13.9 | 1.1 |
|  | Hfp | 15.9 | 3.9 |  | Bio18 | 9.6 | 0.2 |
|  | Bio19 | 9.7 | 10.2 |  | Hfp | 9.6 | 2.9 |
|  | Bio15 | 5.6 | 2.7 |  | Bio4 | 9.3 | 33.1 |
|  | Bio18 | 4.9 | 3.1 |  | Slope | 6.2 | 0.3 |
|  | Awc_class | 3.9 | 0.3 |  | Aspect | 2.3 | 0.5 |
|  | Slope | 3.8 | 0 |  | Awc_class | 1.9 | 0.4 |
|  | T_clay | 1.2 | 0.1 |  | Bio3 | 1 | 13.2 |
|  | Aspect | 0.7 | 0.1 |  | T_ph_h2o | 0 | 0 |
| *F. delavayi* | Elevation | 54 | 79.2 |  |  |  |  |
|  | Hfp | 20.8 | 6.2 |  |  |  |  |
|  | Bio18 | 9.5 | 8.9 |  |  |  |  |
|  | Bio3 | 9.5 | 3.3 |  |  |  |  |
|  | T_ph_h2o | 4.4 | 0.8 |  |  |  |  |
|  | T_clay | 0.7 | 1.1 |  |  |  |  |
|  | Bio19 | 0.6 | 0.5 |  |  |  |  |
|  | Slope | 0.3 | 0 |  |  |  |  |
|  | Aspect | 0.2 | 0.1 |  |  |  |  |
